# Predicting work ability impairment in post COVID-19 patients: a machine learning model based on clinical parameters

**DOI:** 10.1007/s15010-024-02459-8

**Published:** 2025-01-16

**Authors:** Tarek Jebrini, Michael Ruzicka, Felix Völk, Gerardo Jesus Ibarra Fonseca, Anna Pernpruner, Christopher Benesch, Elisabeth Valdinoci, Max von Baum, Martin Weigl, Marion Subklewe, Michael von Bergwelt-Baildon, Julia Roider, Julia Mayerle, Bernhard Heindl, Kristina Adorjan, Hans Christian Stubbe

**Affiliations:** 1 Department of Psychiatry and Psychotherapy, Ludwig Maximilian University (LMU) University Hospital, LMU Munich, Munich, Germany; 2 Department of Medicine III, LMU University Hospital, LMU Munich, Munich, Germany; 3 Department of Medicine IV, LMU University Hospital, LMU Munich, Munich, Germany; 4 Department of Medicine II, LMU University Hospital, LMU Munich, Munich, Germany; 5 German Center for Infection Research, Partner Site Munich, Munich, Germany; 6 Department of Orthopaedics and Trauma Surgery, Physical and Rehabilitation Medicine, Musculoskeletal University Center Munich (MUM), LMU University Hospital, LMU Munich, Munich, Germany; 7 Stabstelle Strategische Unternehmenssteuerung, LMU Munich, Munich, Germany; 8 Department of Psychiatry and Psychotherapy, University of Bern, Bern, Switzerland; 9 Institute of Psychiatric Phenomics and Genomics, LMU University Hospital, Munich, Germany; 10 Department of Medicine III, LMU Klinikum, LMU Munich, Marchioninistrasse 15, 81377 Munich, Germany

**Keywords:** Post COVID-19 syndrome, Long COVID, Work ability, Mental health, Infectious disease, COVID-19, Post COVID

## Abstract

The Post COVID-19 condition (PCC) is a complex disease affecting health and everyday functioning. This is well reflected by a patient’s inability to work (ITW). In this study, we aimed to investigate factors associated with ITW (1) and to design a machine learning-based model for predicting ITW (2) twelve months after baseline. We selected patients from the post COVID care study (PCC-study) with data on their ability to work. To identify factors associated with ITW, we compared PCC patients with and without ITW. For constructing a predictive model, we selected nine clinical parameters: hospitalization during the acute SARS-CoV-2 infection, WHO severity of acute infection, presence of somatic comorbidities, presence of psychiatric comorbidities, age, height, weight, Karnofsky index, and symptoms. The model was trained to predict ITW twelve months after baseline using TensorFlow Decision Forests. Its performance was investigated using cross-validation and an independent testing dataset. In total, 259 PCC patients were included in this analysis. We observed that ITW was associated with dyslipidemia, worse patient reported outcomes (FSS, WHOQOL-BREF, PHQ-9), a higher rate of preexisting psychiatric conditions, and a more extensive medical work-up. The predictive model exhibited a mean AUC of 0.83 (95% CI: 0.78; 0.88) in the 10-fold cross-validation. In the testing dataset, the AUC was 0.76 (95% CI: 0.58; 0.93). In conclusion, we identified several factors associated with ITW. The predictive model performed very well. It could guide management decisions and help setting mid- to long-term treatment goals by aiding the identification of patients at risk of extended ITW.

**Supplementary Information:**

The online version contains supplementary material available at 10.1007/s15010-024-02459-8.

## Introduction

The Post COVID-19 condition (PCC) presents with a broad variety of somatic and psychiatric symptoms [1–6]. The WHO estimates that 10–20% of patients infected with SARS-CoV-2 are affected by a PCC [7]. In some cases, the condition is associated with severe impairment of everyday functioning [4–6, 8, 9]. By definition of the WHO, health is defined through the capacity and motivation of an individual to live an economically and socially active life [10, 11]. In this context, impaired everyday functioning and inability to work (ITW) are indicative of compromised health. Preventive measures and early interventions in PCC patients at risk of ITW can be seen as the core principle of occupational rehabilitation. To select patients at risk of prolonged ITW for early healthcare interventions, predictive tools are urgently needed.

There are various definitions of work ability. In this work, we adhere to the definition by Berg et al. and Lindberg et al., who define work ability as not being on a long-term sick leave [12, 13], which can be measured via work absenteeism. Poor work ability itself is associated with adverse outcomes like impaired mental and physical health (e.g. poor musculoskeletal capacity, obesity, high mental work demands, lack of autonomy [12], and lower overall life satisfaction [14]). In Germany, approximately 10% of COVID-19 patients were unable to work for at least 12 weeks [15, 16]. Every 30th patient suffering from PCC received a partial or full disability pension. Associated costs with disability pensions in Germany were estimated to amount to 2.1 billion euros per year [15], stressing the burden of PCC in economic dimensions.

There are some studies investigating work ability in the context of PCC [17]. According to a systematic review by Gualano et al., the rate of regaining full work ability varies between 10 and 100% depending on the follow-up period, the country of the study, and the severity of the acute infection associated with the PCC [18–21]. Studies looking at a time frame of 6 months found the work inability persisted in 12–43% of patients who were initially unable to work [18, 20, 22]. Risk factors associated with impaired work ability were older age, hospitalization during the acute infection with severe acute respiratory syndrome, higher number of comorbidities, and female sex.

In this study, we sought to identify clinical parameters and biomarkers associated with work ability impairment in PCC patients (1) and to create a predictive model for identifying patients at risk of extended inability to work (2). To this end, we analyzed clinical data and blood samples. While certain laboratory findings characteristic of the PCC have been described (e.g. hyperlipidemia [23–25], compromised coagulation and elevated inflammatory markers [26, 27]), their significance in the context of work ability remains elusive. Based on our findings, we constructed a machine learning (ML) based model based on clinical parameters to predict work ability in PCC patients.

## Patients and methods

### Study inclusion

Patients with PCC as defined by the WHO presenting to the Post COVID-19 outpatient department of the Ludwig Maximilian University (LMU) Munich, Germany, were included into the PCC study if the initial SARS-CoV-2 infection was diagnosed by PCR testing within the past 4–12 months. Written informed consent was obtained from all study participants.

### Medical examination and PROMs at baseline

At the first presentation (referred to by the term “baseline”), patients were examined by an attending physician (internal medicine) and psychiatrist and/or psychologist. Blood was drawn and laboratory tests were conducted according to a standardized protocol in the certified laboratory of the Ludwigs-Maximilians-University hospital. Patients were managed interdisciplinary (e.g. cardiology, pneumology, neurology, endocrinology, …) depending on the individual needs. Each patient answered a series of standardized clinical questionnaires including demographic data, patient reported symptoms, and the following patient-reported outcomes (PROs):

1. WHO Quality of Life Assessment [28], measuring quality of life (QoL) in the four sections physical health, psychological health, social relationship, and environment.
2. 9-item Patient Health Questionnaire [17], a test to screen for symptoms of depression.

Further, the functional performance status was assessed by means of the Karnofsky index [29–31].

### Patient follow-up

Patients were followed up to 12 months after baseline presentation. The follow-up assessments included medical examination by an internist and psychologist or psychiatrist, laboratory tests by the certified laboratory of the university hospital if clinically indicated, and further diagnostic assessments if necessary. In the follow-up visits, patients were asked to answer the clinical questionnaires as described above.

### Data acquisition

Clinical data, PROMs and laboratory parameters were recorded using the lightweight clinical data acquisition and management software for clinical research (LCARS-C, version 1.0, LMU Munich, Germany) [32].

### Statistical analyses

Statistical tests were conducted using R (version 4.3.2). Results for numeric variables are displayed as median values with interquartile ranges (IQR). Results for categorical variables are expressed as absolute numbers/counts with percentages. Medians were compared by use of a two-sided Kruskal-Wallis test. Counts were compared by Pearson’s Chi-squared test. 95% confidence intervals (CI) are shown were applicable. P-values < 0.05 were considered statistically significant. P-values were adjusted if appropriate using the Benjamini-Hochberg procedure. Before the study was started, a prospective power calculation was performed estimating a minimum of 120 patients for this analysis.

### Predictive modelling

We trained a TensorFlow Decision Forests Gradient Boosted Trees Model (TFDF-GB; version 1.9.1) with Python (version 3.11.4) [33, 34] to make predictions on the probability of ITW within the next 12 months in PCC patients. We selected patients with follow-up data at 12 months. Clinical parameters for model training were chosen from the available and standardized assessment in our Post-COVID-clinic. Parameters were selected, if they were readily available in a regular clinical context in order to facilitate model implementation in clinical routine. We removed variables with more than 20% missing values. Next, we excluded patients with more than 20% missing of the remaining variables. We partitioned the dataset in training and test data with 80% for training and 20% for testing. To avoid missingness as a confounder, we imputed the remaining missing values using the multivariate imputer from scikit-learn (version 1.4.2). To balance the minority class (being unable to work), we oversampled the training dataset using the Synthetic Minority Over-sampling Technique (SMOTE) from the Python package imblearn (version 0.12.2) [35, 36]. We trained the TFDF-GN model with 300 trees. We investigated the model’s performance using a 10-fold cross validation. Finally, the model was tested using the testing dataset. The model’s performance was assessed using standard parameters such as receiver-operator characteristics curve and area under the ROC curve (AUC). The performance measures were computed using R (version 4.3.2) [37].

## Results

### Patient demographics (at baseline)

**Fig. 1:**
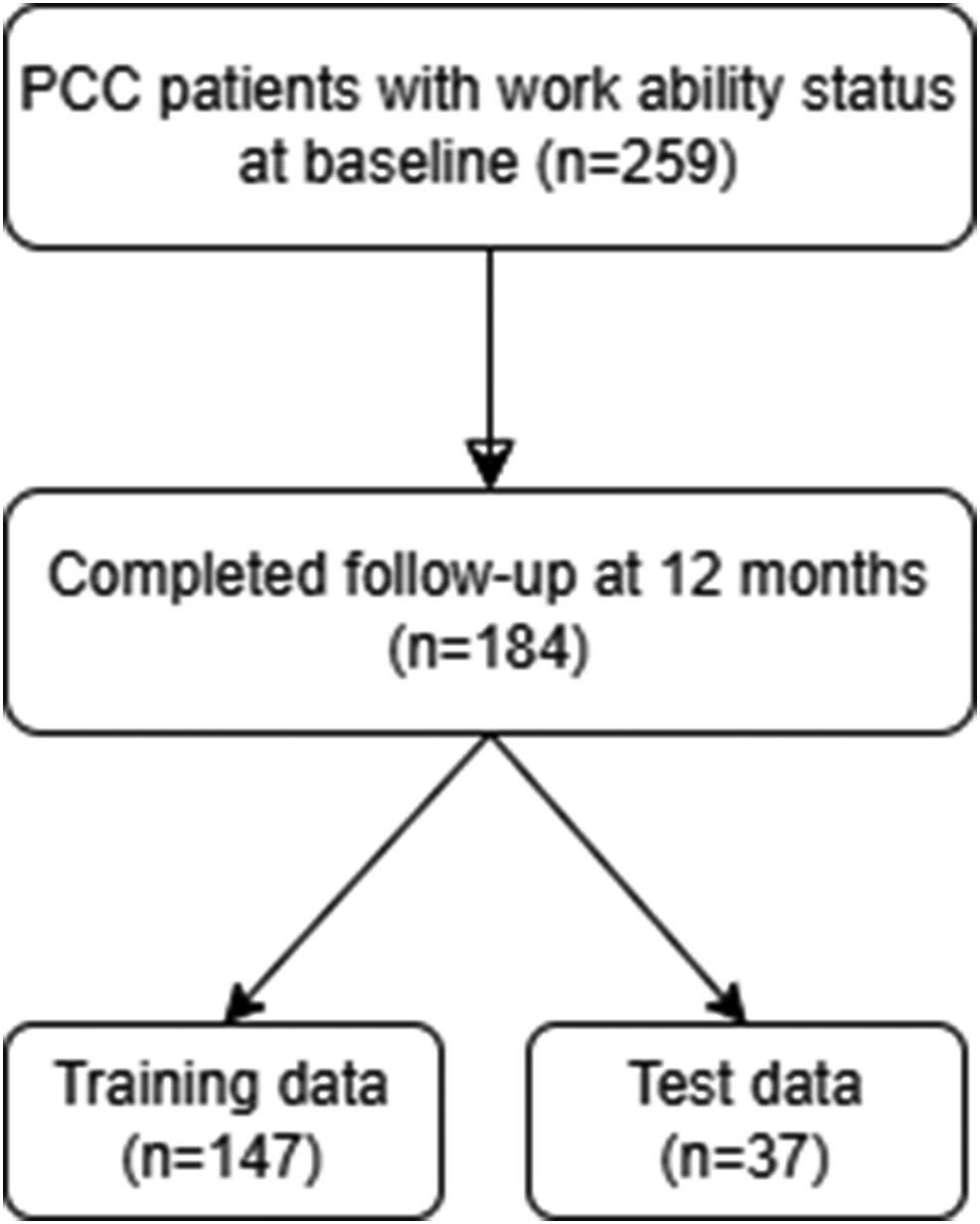
Flow diagram of study inclusion. The figure depicts the inclusion and assessment of patients at baseline and follow-up after 12 months

Of 259 PCC patients above the age of 18 years, *n* = 163 patients reported ITW. Of those, *n* = 99 (60.7%) were female and *n* = 64 (39.3%) were male. The control group consisted of PCC patients who were able to work (ATW; *n* = 96), of which 63 (65.6%) were female and 33 (34.4%) were male (Fig. 1). There was no statistically significant age difference between the two groups (mean age ITW 41.0 vs. ATW 42.0 years). The vast majority of the study population was of European ethnicity (not able to work 100% vs. able to work 99%). The two groups did differ regarding the academic degree to a statistically significant extent. Patients in the ATW group had higher academic degrees (*p* < 0.05) than patients in the ITW group. There was no difference between the two groups regarding marital status, family status, employment, or the field of profession. Demographic data are shown in Table 1; Fig. 1.

**Table 1:** Descriptive patient data and patient-reported outcome measures

Variable	Control group, *N* = 96^1^	ITW group, *N* = 163^1^	*P*-value^2^
**Unable to work**	yes	no	
**Age at inclusion** (years)	42 [31, 54]	41 [33, 52]	n.s.
**Sex at birth** Female Male	63 (66%) 33 (34%)	99 (61%) 64 (39%)	n.s.
Body Weight	70.0 [61.5;80.5]	72.0 [63.0;85.0]	n.s.
Body Height	170 [165;175]	172 [166;180]	n.s.
European Ethnicity	95 (99.0%)	163 (100%)	n.s
**On sick leave** No Unknown Yes	81 (86.%) 8 (8.5%) 5 (5.3%)	21 (15.1%) 5 (3.1%) 130 (81.8%)	< 0.001
**Has psychiatric diagnosis**	19 (19.8%)	61 (37.4%)	0.005
**Has somatic diagnosis**	71 (74.0%)	125 (76.7%)	n.s.
**Unable to work baseline**	0 (0%)	163 (100%)	< 0.001
**PROMs**			
**WHOQOL-BREF: physical health**	53 (39.3;65.2)	39 (25.9;50.0)	0.001
**WHO psychological health** (points)	62 (50.0;70.8)	50 (41.7;66.7)	0.012
**WHO social relationship** (points)	75 (50.0;83.3)	67 (50, 75)	n.s.
**WHO environment** (points)	75 (68.8;84.4)	72 (57.7;78.1)	0.01
**PHQ-9 Score** (points)	8.00 (5.50;11.5)	11.5 (8.00;16.0)	0.002
**Karnofsky index**	80 (80, 90)	70 (70, 70)	< 0.001

### Symptoms and PROMs

Patients of the ITW group stated concentration difficulties (*p* = 0.005), disturbance of appetite (*p* < 0.05), dissatisfaction with the own work ability (*p* < 0.001), and fatigue (*p* < 0.005) more frequently than patients in the ATW group.

Next, we assessed Patient-reported outcome measures (PROMs; Table 1). In the physical, psychological and environmental section of the WHOQOL-BREF, ITW patients scored significantly worse (*p* = 0.001, 0.012 or 0.01, respectively). Fewer ITW patients reported enjoying life (*p* > 0.005)). In line with these findings, patients in the ITW group rate their overall health and quality of life significantly lower (*p* < 0.05) than patients in the control group.

Based on the Karnofsky Index, patients in the ITW group experienced significantly higher impairment of everyday life than patients in the ATW group (*p* > 0.005). ITW patients showed significantly higher levels of depressive symptoms as reflected by the median PHQ-9 scores than ATW patients (*p* = 0.002).

**Fig. 2:**
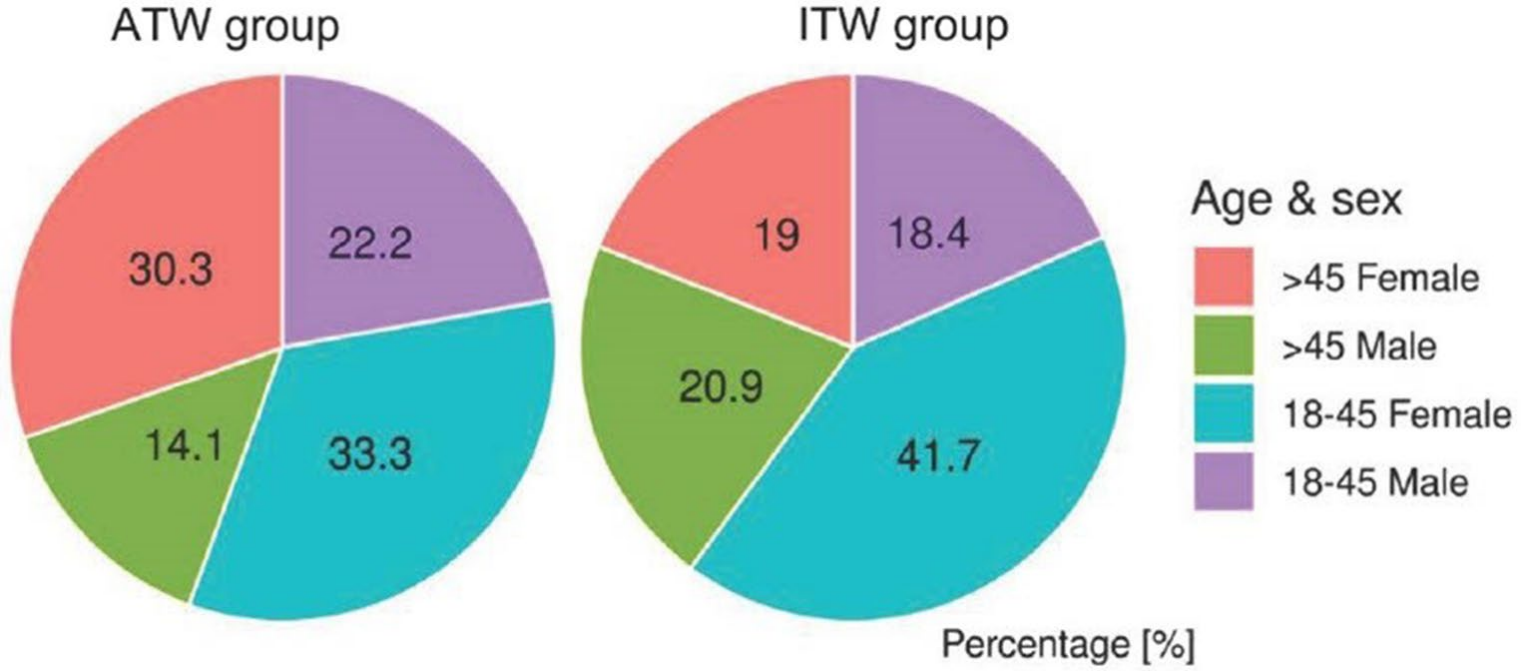
Distribution of sex and age across PCC patients with ITW and ATW respectively. The figure shows the sex and age distribution of each group at baseline

**Fig. 3:**
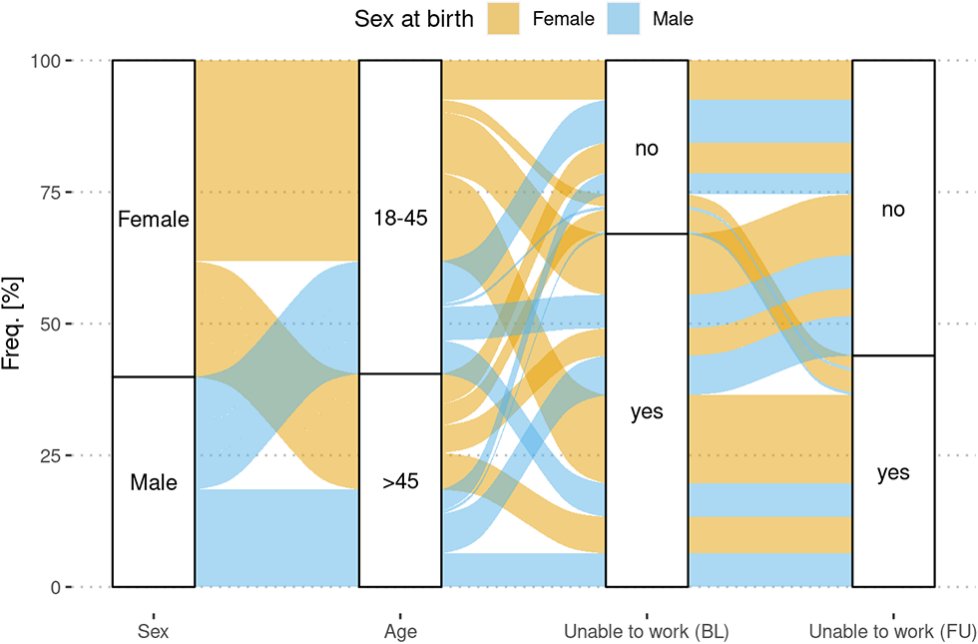
Alluvial diagram of sex, age and ability to work. The diagram reflects the changes of ITW comparing baseline and follow-up

ITW decreased significantly at the follow-up measurement. Changes in the distribution between the groups regarding ITW by gender and age are shown in Figures 2 and 3.

### Laboratory measures

**Fig. 4:**
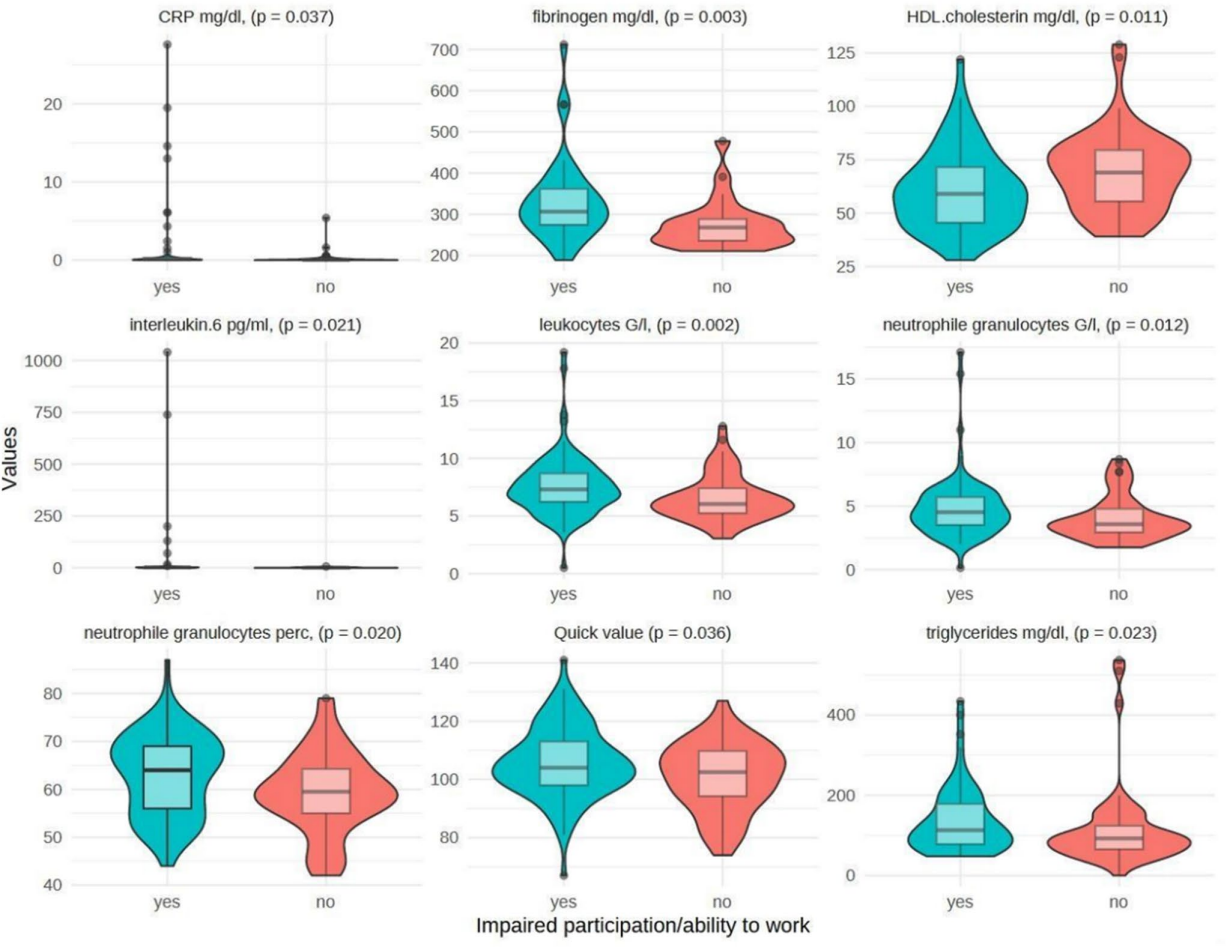
Values of laboratory parameters in related unit by target group (yes) versus control group (no)

Laboratory findings with statistically significant differences between the two groups are depicted in Fig. 4.

We found elevated inflammatory blood serum parameters in ITW patients, namely CRP (*p* < 0.05), interleukin-6 (*p* < 0.05), leukocytes (6.9, *p* < 0.05) and neutrophile granulocytes (*p* < 0.05). Further, parameters of coagulation in the blood serum differed significantly between the groups. In the ITW group, higher Quick Values (*p* < 0.05) and higher levels of fibrinogen (*p* = 0.003) were found. Differences between the groups regarding parameters of dyslipidemia such as lower levels of HDL-Cholesterol (*p* < 0.05) and higher levels of triglycerides (*p* < 0.05) were found in the ITW group.

### Predicting inability to work

**Fig. 5:**
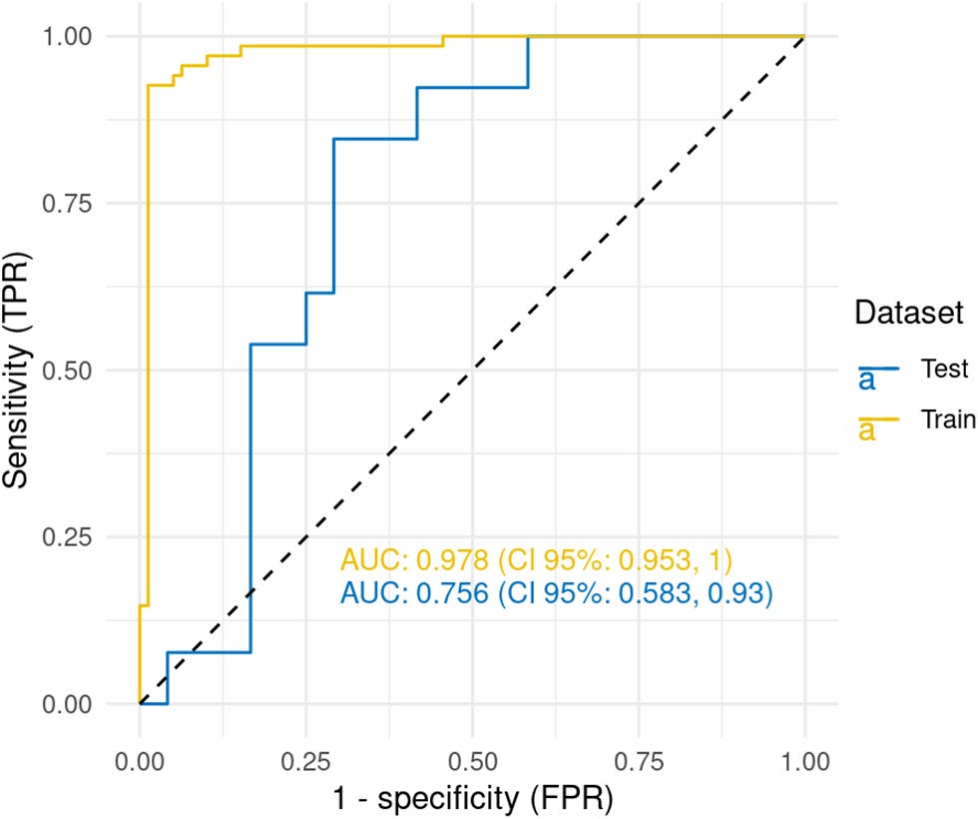
TFDF-GB model ROC curve and AUC. The figure shows the ROC curves for the predicted outcome of inability to work at 12 months after baseline together with the respective AUC. The x-axis is 1 - specificity (false positive rate– FPR). The y-axis shows the sensitivity (true positives rate– TPR). The data includes the ROC curves for training data (yellow) and testing data (blue)

For training the model, we selected patients with severe PCC and completed follow-up data at 12 months (*n* = 184). The dataset was split for model training (*n* = 147) and testing (*n* = 37). To avoid missingness as training parameter, we removed patients with high rates of missing values (> 20% missing) reducing the training dataset from 147 to 141 patients. Next, we imputed missing values in the training datasets using the multivariate imputer of the scikit-learn package. We addressed the imbalance in the outcome parameter (ITW at 12 months), by up-sampling our training dataset to a total of 228 samples using SMOTE. The following two parameters of the acute infection were included in the model: hospitalization and WHO severity of infection. From the baseline data, the following seven parameters were selected: symptoms at baseline, presence of somatic diagnoses, presence of psychiatric diagnoses, age, height, weight, and Karnofsky index (see Supplementary Table 1). A TFDF-GB model with 300 trees was trained to predict inability to work 12 months after baseline. TFDF-GB sequentially builds an ensemble of decision trees to enhance prediction accuracy. It is compute-efficient and does not require normalization of the training data. Its robustness has been confirmed in previous studies making it an ideal choice for our study [1]. We trained the model with 300 trees, which is considered a reliable standard. Using a 10-fold cross-validation, the model performance was estimated at a mean AUC of 0.83 (95% CI: 0.78; 0.88). In the testing data and given the challenging scenario, the model exhibited an AUC of 0.76 (95% CI: 0.58; 0.93, Fig. 4). The sensitivity of the model was 0.58 (95% CI: 0.35; 0.81) and the specificity was 0.89 (95% CI: 0.75; 1.00). The positive predictive value (PPV) of the model was 0.87 (95% CI: 0.69; 1.00). The model’s negative predictive value (NPV) was 0.61 (95% CI: 0.42; 0.81). The most important model feature was the Karnofsky index at baseline followed by body height, body weight, age at baseline, and features derived from the symptom variable at baseline (see Supplementary figure S1). An exemplary decision tree is depicted in Supplementary figure S2. Model metrics over different cut-offs are shown in Supplementary figure S3 (See Fig. 5).

## Discussion

ITW is a severe adverse outcome of PCC. In this study, we found clinical and laboratory factors associated with ITW. Based on these findings, we trained a TFDF-GB model for identifying patients at risk of ITW 12 months after baseline.

Inflammation biomarkers were mildly but significantly elevated in the ITW group. These findings may support the hypothesis that subclinical, prolonged inflammatory responses could contribute to PCC [26]. Alternatively, a lack of physical activity might exert a negative impact on these biomarkers.

Similarly, we observed compromised coagulation parameters compared to the ATW group. In line with our findings, a meta-analysis by Korompoki et al. described changes in coagulation parameters of PCC patients as frequent across the published literature [38]. They hypothesized that these changes could be attributed to immunothrombotic processes, which may be more pronounced in severe cases of PCC. Alternatively, the differences in laboratory parameters could be attributed to reduced physical activity and its negative consequences on various organ systems [39]. Similar findings in patients with chronic fatigue syndrome (CFS) are well established [40–42]. Reduced physical activity is a known risk factor for the development of dys- and hyperlipidaemia [43, 44]. These observations are further supported by the hyperlipidaemia we observed in many of our ITW patients.

The laboratory factors reported above were altered significantly but with very small effect sizes. Therefore, and to keep the model more simple, the laboratory factors were not included in the TFDF-GB model. Follow-up studies should aim to deepen insight into the relationship between the changes in parameters of inflammation, hyperlipidemia and anticoagulation with PCC and ITW.

PCC patients with ITW stated higher levels of fatigue which is associated with reduced physical activity. The central role of fatigue in PCC symptomatology is well described in the published literature [45–47], and the association of fatigue with ITW has been demonstrated [46, 48–50]. Addressing fatigue in PCC could be a highly relevant target for improving or preventing ITW in PCC patients.

Psychiatric diagnoses (e.g. depression) are risk factors for the development of PCC [48, 51]. We found a correlation between psychiatric symptoms and ITW, which is in line with findings by Kerksiek et al. [17]. In the ITW group, patients reported significantly lower QoL regarding physical and mental health and their environment. This observation corresponds to previous studies [52–54]. Additionally, ITW patients reported finding their lives less meaningful. They also experienced dissatisfaction with their working ability, concentration difficulties, inappetence, and loss of energy more frequently than PCC patients without ITW. These observations support the hypothesis that PCC patients with ITW suffer from PCC of higher severity than those without ITW. Importantly, long-term ITW by itself has a negative influence on QoL as demonstrated in other patient cohorts [55, 56].

From the above parameters, we designed the TFDF-GB model. Given the challenging prediction, the model performed quite well: the model’s AUC of 0.76 (95% CI: 0.58; 0.93) was more than acceptable in the testing dataset, while being excellent in the cross-validation. The model’s PPV at the selected cut-off was high with 0.87 (95% CI: 0.69; 1.00) and suited to identify patients at risk of prolonged ITW.

A limitation of our study is that many relevant constructs (e.g. QoL) were assessed via PROs, which are prone to subjective bias (e.g. recall bias) [57]. However, PROs are valuable tools to depict the perceived reality of individuals affected. One important limitation is the European ethnicity of the study population, which limits the generalization to the European clinical context. Further, the investigated sample consisted of patients of a university hospital, possibly selecting a more severe or persistent disease progression. Another limitation is that reasons for impaired work ability were only partially assessed. Future studies should address the tasks PCC patients are unable to carry out in more detail and inspect the reasons for ITW in more detail. This would enable new strategies for job reintegration specific for PCC patients. In follow-up studies, work ability should be assessed as a multidimensional concept. In this regard, measures like job productivity, disabilities at work, and perceived work ability could be included to generate a more precise and gradual measurement of the ability to work. Lastly, Machine learning (ML) models bear the risk of overfitting. In contrast to neural networks and other deep learning models, decision forests, such as the TFDF-GB model, are regarded as less susceptible to overfitting, robust to missing values, and suitable for training in smaller datasets. Still, further external validation of the established model is needed for improving the model’s generalization ability.

Taken together, our data demonstrate that PCC substantially affects the workforce. ITW in PCC is significantly associated with fatigue, reduced QoL, and changes in lipid metabolism, inflammatory, and blood coagulation parameters. Based on these findings, we established the first ML model to predict ITW in PCC patients. Identifying patients at risk is crucial to establish preventive measures and early interventions with the goal of occupational rehabilitation [17]. Even though the clinical, economic, and social burden of ITW in PCC is enormous, this is the first published predictive approach on ITW in PCC to the knowledge of the authors. We believe that our model might guide preventive measures in PCC patients at risk of ITW and could be implemented to design personalized clinical rehabilitation programs for the patient at risk of an extended ITW. Future studies should test our ML model in larger and independent PCC patient cohorts.

## Electronic supplementary material

Below is the link to the electronic supplementary material.

Supplementary Material 1

Supplementary Material 2

Supplementary Material 3

Supplementary Material 4

## Data Availability

Data will be provided on request.

## Declarations

### Trial registration number & date of registration

DRKS00030974, 22 Dec 2022.
