# Supplementary material for: Predicting work ability impairment in post COVID-19 patients: a machine learning model based on clinical parameters
Source: Infection. 2025 Jan 16;53(3):1189–97. doi: 10.1007/s15010-024-02459-8 (PMC12137377; doi:10.1007/s15010-024-02459-8)
Supplement: Supplementary file 1 — Supplementary Material 1 [file 15010_2024_2459_MOESM1_ESM.docx]

| **Supplementary table 1. Variables and features for the TFDF model.** | | | |
| --- | --- | --- | --- |
| **Variable** | **Feature name** | **Type** | **Timepoint** |
| hospitalization | hospitalization | Categorical | acute infection |
| infection severity (WHO) | infection severity (WHO) | Numeric | acute infection |
| symptoms | abdominal pain | Categorical | baseline |
| symptoms | sweating | Categorical | baseline |
| symptoms | chest pain | Categorical | baseline |
| symptoms | confusion | Categorical | baseline |
| symptoms | congestion runny nose | Categorical | baseline |
| symptoms | constipation | Categorical | baseline |
| symptoms | cough | Categorical | baseline |
| symptoms | diarrhea | Categorical | baseline |
| symptoms | dizziness | Categorical | baseline |
| symptoms | dysphagia | Categorical | baseline |
| symptoms | dyspnea | Categorical | baseline |
| symptoms | dysuria | Categorical | baseline |
| symptoms | eye sight disorder | Categorical | baseline |
| symptoms | fatigue | Categorical | baseline |
| symptoms | fever | Categorical | baseline |
| symptoms | hair loss | Categorical | baseline |
| symptoms | headache | Categorical | baseline |
| symptoms | palpitations | Categorical | baseline |
| symptoms | hematemesis | Categorical | baseline |
| symptoms | hematochezia | Categorical | baseline |
| symptoms | hemoptysis | Categorical | baseline |
| symptoms | hpoesthesia | Categorical | baseline |
| symptoms | impaired alertness | Categorical | baseline |
| symptoms | imparied speech | Categorical | baseline |
| symptoms | insomnia | Categorical | baseline |
| symptoms | joint or muscle pain | Categorical | baseline |
| symptoms | smell loss | Categorical | baseline |
| symptoms | taste loss | Categorical | baseline |
| symptoms | melena | Categorical | baseline |
| symptoms | memory impairment | Categorical | baseline |
| symptoms | nausea | Categorical | baseline |
| symptoms | night sweats | Categorical | baseline |
| symptoms | paresis paralysis | Categorical | baseline |
| symptoms | peripheral edema | Categorical | baseline |
| symptoms | red eye | Categorical | baseline |
| symptoms | reduced muscular strength | Categorical | baseline |
| symptoms | throatache | Categorical | baseline |
| symptoms | tremor | Categorical | baseline |
| symptoms | impaired stand | Categorical | baseline |
| symptoms | impaired walk | Categorical | baseline |
| symptoms | unilateral leg pain | Categorical | baseline |
| symptoms | unilateral swollen leg | Categorical | baseline |
| symptoms | unwanted weight loss | Categorical | baseline |
| symptoms | other | Categorical | baseline |
| symptoms | paresthesia | Categorical | baseline |
| symptoms | anxiety or strain | Categorical | baseline |
| symptoms | tinnitus | Categorical | baseline |
| symptoms | depressive mood | Categorical | baseline |
| symptoms | vertigo | Categorical | baseline |
| symptoms | vomiting | Categorical | baseline |
| has psychiatric diagnosis | has psychiatric diagnosis | Categorical | baseline |
| has somatic diagnosis | has somatic diagnosis | Categorical | baseline |
| age | age at inclusion | Numeric | baseline |
| body weight | body weight | Numeric | baseline |
| body heigth | body heigth | Numeric | baseline |
| karnofsky index current | karnofsky index current | Numeric | baseline |
