# Supplementary material for: Predicting work ability impairment in post COVID-19 patients: a machine learning model based on clinical parameters
Source: Infection. 2025 Jan 16;53(3):1189–97. doi: 10.1007/s15010-024-02459-8 (PMC12137377; doi:10.1007/s15010-024-02459-8)
Supplement: Supplementary file 2 — Supplementary Material 2 [file 15010_2024_2459_MOESM2_ESM.pdf]

Supplementary figure S2

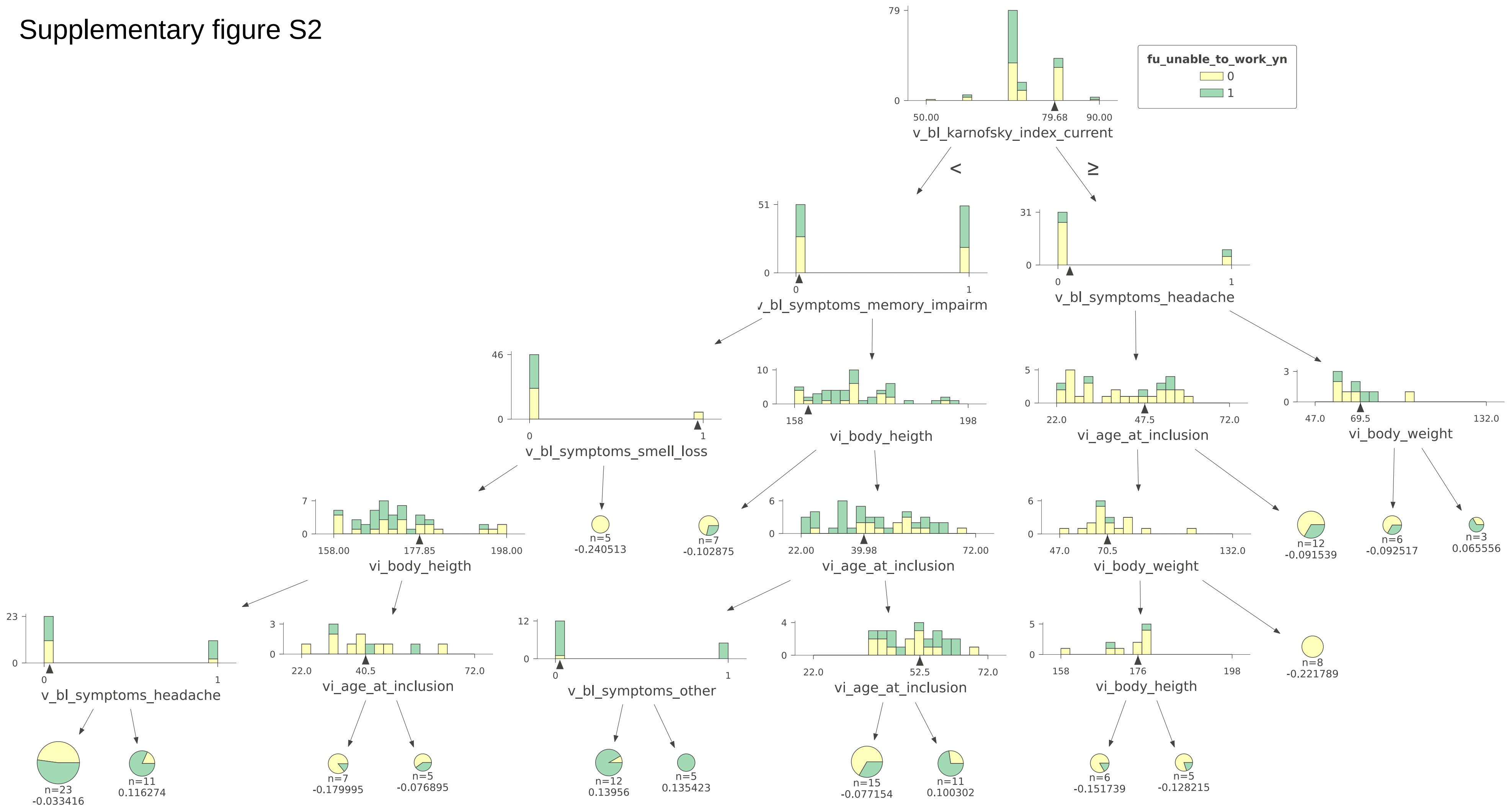

Supplementary figure S2. Exemplary decision tree of the TFDF-GB model. The model was trained with 300 trees. This figure depicts all levels of an exemplary decision tree.
