# Supplementary figures and images for: Predicting work ability impairment in post COVID-19 patients: a machine learning model based on clinical parameters
Source: Infection. 2025 Jan 16;53(3):1189–97. doi: 10.1007/s15010-024-02459-8 (PMC12137377; doi:10.1007/s15010-024-02459-8)

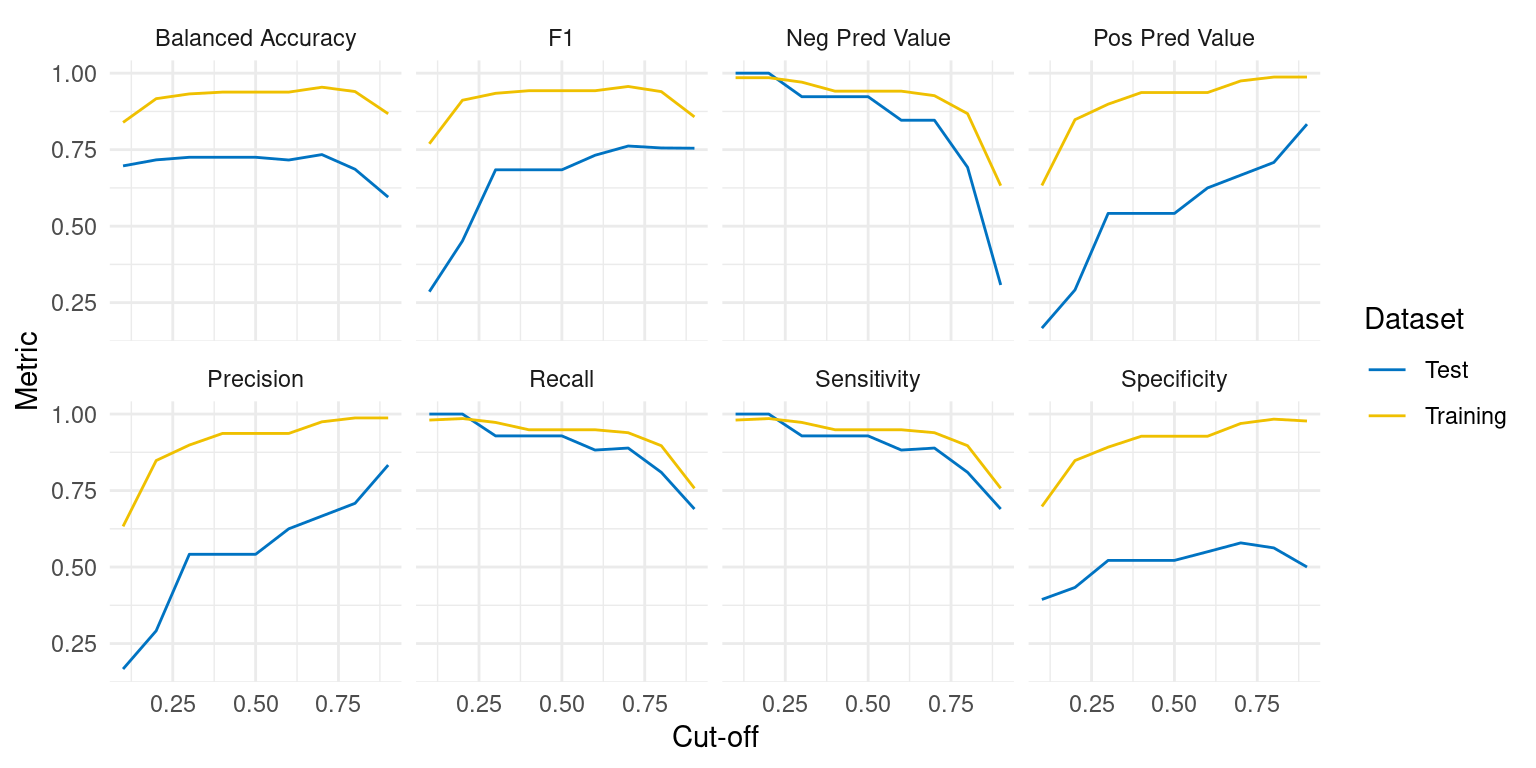

Supplement: Supplementary file 4 — Supplementary Material 4 [file 15010_2024_2459_MOESM4_ESM.png]
